# Canthaxanthin Biofabrication, Loading in Green Phospholipid Vesicles and Evaluation of In Vitro Protection of Cells and Promotion of Their Monolayer Regeneration

**DOI:** 10.3390/biomedicines10010157

**Published:** 2022-01-12

**Authors:** Ines Castangia, Maria Letizia Manca, Seyed Hadi Razavi, Amparo Nácher, Octavio Díez-Sales, José Esteban Peris, Mohamad Allaw, Maria Carmen Terencio, Iris Usach, Maria Manconi

**Affiliations:** 1 Department Scienze della Vita e dell’Ambiente, University of Cagliari, 09124 Cagliari, Italy; inescastangia@tiscali.it (I.C.); allaw.mohamad.22@gmail.com (M.A.); manconi@unica.it (M.M.); 2 Bioprocess Engineering Laboratory (BPEL), Department of Food Science, Engineering & Technology, Faculty of Agricultural Engineering and Technology, University of Tehran, P.O. Box 4111, Karaj 31587-77871, Iran; srazavi@ut.ac.ir; 3 Department of Pharmacy and Pharmaceutical Technology, University of Valencia, 46100 Valencia, Spain; amparo.nacher@uv.es (A.N.); octavio.diez@uv.es (O.D.-S.); Jose.E.Peris@uv.es (J.E.P.); Carmen.Terencio@uv.es (M.C.T.); iris.usach@uv.es (I.U.)

**Keywords:** Dietzia natronolimnaea HS-1, macrophages, fibroblasts, hydrogen peroxide, Griess reagent, canthaxanthin, skin delivery

## Abstract

In the present study, canthaxanthin was produced by biofermentation from Dietzia natronolimnaea HS-1 (D. natronolimnaea) and was loaded in phospholipid vesicles prepared with natural component using an easy and low dissipative method. Indeed, glycerosomes, hyalurosomes, and glycerohyalurosomes were prepared by direct hydration of both phosphatidylcholine and the biotechnological canthaxanthin, avoiding the use of organic solvents. Vesicles were sized from 63 nm to 87 nm and highly negatively charged. They entrapped a high number of the biomolecules and were stable on storage. Canthaxanthin-loaded vesicles incubated with fibroblasts did not affect their viability, proving to be highly biocompatible and capable of inhibiting the death of fibroblasts stressed with hydrogen peroxide. They reduced the nitric oxide expression in macrophages treated with lipopolysaccharides. Moreover, they favoured the cell migration in an in vitro lesion model. Results confirmed the health-promoting potential of canthaxanthin in skin cells, which is potentiated by its suitable loading in phospholipid vesicles, thus suggesting the possible use of these natural bioformulations in both skin protection and regeneration, thanks to the potent antioxidant, anti-inflammatory and antiageing effects of canthaxanthin.

## 1. Introduction

Carotenoids are tetraterpene pigments that are yellow, orange, red, and purple coloured and naturally occurring in the chloroplasts of photosynthetic bacteria and several species of archaea and fungi, algae, plants, and animals [1]. Indeed, bacteria, fungi, algae, and plants can synthesize carotenoids, while animals cannot, and their supply can be ensured by the intake of food and, sometimes, their metabolic modification [1]. Carotenoids play important roles in animals, as they are precursors of vitamin A, such as photoprotectors, antioxidants, anti-inflammatories, enhancers of immunity, and contributors to reproduction; thus, they are necessary to maintain normal health and behaviour. Given that, they are extensively used as colourants and functional molecules in the food, cosmetic, and pharmaceutical industries [2,3]. They are among the most abundant pigments in nature (more than 750) and are classified into two main groups: carotenes and xanthophylls [4]. Carotenes are hydrocarbons only containing carbon and hydrogen atoms, and the most abundant are α-carotene, β-carotene, γ-carotene, and lycopene. Xanthophylls are carotenoids containing hydroxyl, carbonyl, aldehyde, carboxylic, epoxide, and furan-oxide groups and include lutein, cryptoxanthin, zeaxanthin, astaxanthin, and canthaxanthin. Canthaxanthin (4,4′-diketo-β-carotene) is a diketocarotenoid containing nine conjugated double-bonds, terminated by two oxo substituents. Thanks to its peculiar structure, canthaxanthin is intensely coloured (orange-red) and has strong free radical scavenging properties [5]. It is naturally present in bacteria, algae, and some fungi and is responsible for the intense colour of several animals, such as flamingos, mushrooms, and crustacea; it was detected for the first time in *Cantharellus cinnabarinus* [6]. Canthaxanthin is not a vitamin A precursor but an intermediate product obtained during the metabolism from β-carotene to astaxanthin and, in animals, can address beneficial biological activities, mostly related to its antioxidant and scavenging abilities, thus preventing skin ageing and some other diseases by favouring tissue regeneration and strengthening the immune system [7]. It can be obtained from natural sources [5], by total synthesis, or, alternatively, by bioproduction using specific microorganisms, among which *D. natronolimnaea*, a Gram-positive, catalase-positive, and oxidase-negative bacterium, is considered the favourite [8,9]. The bioproduction addresses an inexpensive and advanced biotechnological method, not seasonally or geographically dependent, which avoids the depauperating of natural resources and pollutant chemical synthesis, thus providing a satisfying alternative at the industrial level. Moreover, *D. natronolimnaea* can use molasses as inexpensive and renewable substrates in place of glucose to greatly produce canthaxanthin [10]. The promising health-promoting effects and the possibility of using a scalable and sustainable bioproduction of canthaxanthin, make it an ideal candidate to produce natural-based products. Nevertheless, its actual exploitation in commercial products is limited by its instability, due to the unsaturated bonds and its lipophilic nature, which make it poorly soluble in biological fluids and less bioavailable. The loading in suitable nanocarriers has been proposed as an effective alternative to ameliorate canthaxanthin in vivo availability and effectiveness [11,12]. Among nanocarriers, advanced phospholipid vesicles represent an ideal choice when natural and environmentally friendly products must be manufactured, given the natural origin of phospholipids and their easy and low dissipative preparation methods [13]. In particular, they are optimal carriers for skin delivery, which improve the deposition and residence time in the deeper skin layer [14,15,16,17,18].

In the present study, aiming at manufacturing a biotechnological, green, and natural formulation for skin delivery, canthaxanthin was loaded in phospholipid vesicles (glycerosomes, hyalurosomes, or glycerohyalurosomes), and their main physicochemical characteristics and technological properties were evaluated along with the in vitro biological ones: the cell biocompatibility, ability to protect fibroblasts from oxidative damages and to inhibit nitric oxide expression, and capability of promoting proliferation and migration of cells, in vitro, in a scratched monolayer.

## 2. Materials and Methods

### 2.1. Materials

Soy lecithin and glycerol were purchased from Galeno (Potenza, Italy). Sodium hyaluronate was purchased from Pentapharm DSM Nutritional Products AG (Aesch/Switzerland). All the chemical products and solvents of analytical grade were purchased from Sigma-Aldrich (Milan, Italy). Cell medium, foetal bovine serum, penicillin, streptomycin, and all the other reagents and plastic for cell culture were purchased from Life Technologies Europe (Monza, Italy).

### 2.2. Bioproduction and of Canthaxanthin

The strain of bacterium *D. natronolimnaea* HS-1 (DSM44860) was maintained on yeast/malt agar plates containing glucose (10 g/L), peptone (5 g/L), yeast extract (5 g/L), malt extract (3 g/L), and agar (15 g/L) at 4 °C and pH 7.31. Molasses diluted at various concentrations (20 g/L), to discard insoluble materials, were used as carbon source substrate. Single colonies were transferred to a fresh plate of yeast/malt agar, incubated for 4 days at 28 ± 1 °C, and kept under refrigeration at 4 °C. After inoculating with colonies of *D. natronolimnaea* HS-1, Erlenmeyer flasks containing glucose (10 g/L), peptone (10 g/L), and yeast extract (6 g/L) were incubated in an orbital incubator (model 96 Stuart S150; Staffordshire, UK) at 180 rpm and 28 ± 2 °C for 6 days to produce canthaxanthin [19].

After the fermentation process, aliquots of obtained media (10 mL) were centrifuged at 7500× *g* for 7.5 min at 4 °C. The pellets were resuspended 3 times in absolute ethanol (3 mL), vortexed for 5 min, and centrifuged again until the pellets were colourless. The supernatant solution obtained was adjusted to pH 3.0 with 0.5 M H_2_SO_4_, left to stand for 24 h at room temperature, and then centrifuged at 10,000× *g* for 10 min. The supernatant was treated with activated carbon without heating to avoid hydrolysis of the sucrose, filtered with 0.2 mm filter paper under vacuum, and finally freeze-dried to obtain a powder.

### 2.3. Canthaxanthin Analysis

A Knauer (Berlin, Germany) HPLC system equipped with a UV–vis detector (K-2600, Knauer, Germany) was used to identify and quantify canthaxanthin [20]. The separation was performed by using a Lichrospher 100 RP-18 silica column (5.0 mm, 250 mm × 4 mm) at 35 °C. The mobile phase was a mixture of acetonitrile, methanol, and dichloromethane (71:22:7, *v*/*v*/*v*) isocratically eluted at a flow rate of 2 mL/min. The volume of solution injected was 10 µL. A pre-column of the same material to protect the column was used as well. An Applied Biosystems API 150 EX (PE Sciex, Toronto, Canada) single quadrupole mass spectrometer equipped with an atmospheric pressure chemical ionisation interface and an Apple Macintosh System v.7.6.1 were used to analyse the tandem mass spectrometric peaks. The data acquisition and processing were performed using a Masschrom v.1.1 application version. The optimum conditions of the interface were: OR voltage 10 V, ring voltage 175 v, Needle Current (NC) 2 μA, curtain gas 10, and nebulising gas 12. The temperature of APCI vaporisation was maintained at 400 °C. The atmospheric pressure chemical ionisation was employed in the positive ionisation mode based upon the structure and the chemical nature of isoprenoids. Mass spectra were obtained over the scan m/z 350–700 range using a step size of 0.2 amu and a dwell time of 0.6 ms.

### 2.4. Ability of Canthaxanthin to Scavenge Free Radicals

The antioxidant potential of canthaxanthin was assessed by measuring its ability to scavenge the DPPH (1,1-diphenyl-1-picrylhydrazil) radicals. The canthaxanthin ethanolic solution (1 mg/mL) was firstly diluted 1:50 to reduce the colour of the solution and then 20 μL of the diluted solution was mixed with 1980 μL of DPPH methanolic solution (40 µg/mL) and incubated for 30 min at room temperature in the dark. Then, the absorbance was measured at 517 nm against blank. The antioxidant activity was calculated according to the following formula:

antioxidant activity (%) = [(ABS_DPPH_ − ABS_sample_)/ABS_DPPH_] × 100

A calibration curve using Trolox (6-hydroxy-2,5,7,8-tetramethylchroman-2-carboxylic acid) at different concentrations (0–0.010 mg/mL) was built and used as reference and expressed as mg of Trolox equivalent/g of dry powder. All the experiments were performed in triplicate.

### 2.5. Vesicle Preparation

Soy lecithin (120 mg/mL) and canthaxanthin (20 mg/mL) were weighed in a glass vial and hydrated with water to prepare liposomes; water and glycerol (30:70) to prepare glycerosomes; water containing sodium hyaluronate (0.1% *p/v*) to prepare hyalurosomes; and water containing sodium hyaluronate solution (0.1% *p/v*) and glycerol (30:70) to obtain glycerohyalurosomes (Table 1) [16,20,21]. Dispersions were left hydrating for 2 h to promote the swelling of the phospholipid and then sonicated (5 s on and 2 s off, 3 cycles; 13 microns of probe amplitude) with a high intensity ultrasonic disintegrator (Soniprep 150, MSE Crowley, London, UK) to obtain small and homogenous vesicles. Empty formulations were also prepared and used as references.

### 2.6. Vesicle Characterisation

The average diameter and polydispersity index of the vesicles were determined by photon correlation spectroscopy using a Zetasizer ultra (Malvern Instruments, Worcestershire, UK). The Zetasizer ultra was also used to measure the surface charge of vesicles (zeta potential) measuring their electrophoretic mobility in dispersion with the mixed-mode measurement-phase analysis (M3-PALS). Each sample was diluted (1:1000) to be optically clear and avoid the attenuation of the laser beam by the particles along with the reduction of scattered light that can be detected.

The entrapment efficiency was calculated as the percentage of antioxidant activity of vesicle dispersions measured before and after their purification from the unentrapped canthaxanthin by means of dialysis method. To this, the vesicle dispersions (1 mL) were loaded in dialysis tube (Spectra/Por^®^ membranes, 12–14 kDa MW cut-off, and 3 nm pore size; Spectrum Laboratories Inc., DG Breda, the Netherlands) and maintained at room temperature in two litres of water for 2 h, refreshing water after 1 h. The antioxidant activity of dispersions before and after the dialysis process was measured by means of the DPPH colorimetric test.

The stability of the vesicles was evaluated monitoring the mean diameter, polydispersity index, zeta potential, and entrapment efficiency over three months of storage at room temperature (25 ± 2 °C).

### 2.7. Ability of Canthaxanthin Formulations to Inhibit the Generation of Nitric Oxide in Macrophages

Macrophages RAW264.7 (2 × 10^5^ cells/well) were preincubated for 1 h with canthaxanthin (in dispersion or loaded in vesicles) appropriately diluted to reach 20 μg/mL, 2 μg/mL, 0.2 μg/mL, and 0.02 μg/mL of payload. The lipopolysaccharide (1 μg/mL, final concentration) was added to each well, and cells were incubated for 20 h at 37 °C. Cells stressed with lipopolysaccharide were used as negative control, and healthy cells unstressed with lipopolysaccharide and untreated with canthaxantin were used as positive control. After incubation, the cell culture medium (100 μL) was withdrawn, and the mixture was transferred into a new 96-well plate and mixed with Griess reagent solution (100 μL). After 5 min of incubation at 25 °C in the dark, the absorbance was measured at 540 nm by using a microplate reader. A standard curve was built by using sodium nitrite solutions in the culture media (0 to 100 μM) and used to calculate the amount of nitrite in the used medium. The amount of produced nitrite was calculated as percentage between the nitrite released in cells treated with samples minus nitrite released in health cells (positive control) versus nitrite released in negative control cells (stressed with lipopolysaccharide only) minus nitrite released in healthy cells (positive control). Then, positive control (healthy cells) corresponds to 0% of nitrite release, and negative control (stressed cells) corresponds to 100% of nitrite release.

### 2.8. In Vitro Cytotoxicity of Formulations

Primary mouse embryonic fibroblasts (3T3) (ATCC collection, Virginia, VA, USA) were grown as monolayer in 75 cm^2^ flasks at 37 °C in 100% humidity and 5% CO_2_ by using phenol red-free Dulbecco’s Modified Eagle’s medium (DMEM) with high glucose, enriched with foetal bovine serum (10% *v/v*) and penicillin-streptomycin. Cells were seeded into 96-well plates (7.5 × 10^3^ cells/well) and, after 24 h, treated for 48 h with canthaxanthin in dispersion (positive control) or loaded into the vesicles. Samples were diluted in cell medium to reach different canthaxanthin concentrations (20 µg/mL, 2 µg/mL, 0.2 µg/mL, and 0.02 µg/mL). At the end of the experiments, the cells were washed three times with warmed PBS, and their viability was measured by using the MTT [3(4,5-dimethylthiazolyl-2)-2, 5-diphenyltetrazolium bromide] colorimetric assay. MTT solution (100 µL 0.5 mg/mL in PBS, final concentration) was added to each well, and cells were incubated for 2–3 h. After that, the formed formazan crystals were dissolved in dimethyl sulfoxide, and their concentration was spectrophotometrically quantified at 570 nm by using a microplate reader (Synergy 4, Reader BioTek Instruments, AHSI S.P.A, Bernareggio, Italy). Results are shown as percent of cell viability in comparison with nontreated cells (100% viability).

### 2.9. In Vitro Protective Effect of Formulations against Oxidative Damage in Fibroblasts

The ability of canthaxanthin to protect fibroblasts from damages induced by hydrogen peroxide was evaluated. Cells were seeded in 96-well plates and incubated at 37 °C in 5% CO_2_ for 24 h. Cells were stressed with hydrogen peroxide (30% diluted 1:40,000 *v*/*v* with PBS) and treated for 4 h with canthaxanthin in dispersion or loaded in vesicles opportunely diluted to reach 2 mg/mL and 0.2 mg/mL of payload. Cells stressed with hydrogen peroxide and untreated were used as negative control, and healthy cells unstressed and untreated were used as positive control. At the end, the cells were washed with PBS, and the MTT assay was used to assess the viability. Untreated cells were used as 100% of viability, and cells stressed with hydrogen peroxide were used as negative control.

### 2.10. In Vitro Scratch Assay

The ability of canthaxanthin to stimulate cell proliferation and migration was evaluated measuring the speed rate of the wound healing as a function of the time of exposition with the samples (scratch assay). Cells were cultured in 6-well plates until the complete confluence was reached. Then, a linear scratch was generated with a sterile plastic pipette tip. The cells were gently washed with fresh medium to remove the scattered fragments and then treated for 48 h with canthaxanthin in dispersion or loaded in vesicles, properly diluted with medium to reach 2 µg/mL and 0.2 µg/mL of payload. Untreated cells were used as positive control. The changes of the area of the lesion were monitored by using an optical microscope (10 × objective), and the related images were captured at initial time to measure the wounded area immediately after scratching (a_0_) and at 24 h and 48 h (a_t_) to measure the areas during the treatment. The captured images were quantified by Java’s image J software (http://rsb.info.nih.gov (accessed on 2 January 2022)) by measuring the area of the wound The migration of cells towards the wounds was expressed as percentage of wound closure:

Wound closure% = [(a_0_ − a_t_/a_0_] × 100%

### 2.11. Statistical Analysis of Data

Results are expressed as the mean ± standard deviation. Analysis of variance (ANOVA) was used for multiple comparisons of means, and Tukey’s test and Student’s *t*-test were performed to substantiate differences between groups using XL Statistics for Windows. The differences were considered statistically significant for *p* < 0.05.

## 3. Results

### 3.1. Canthaxanthin Bioproduction and Quantification

**Figure 1:**
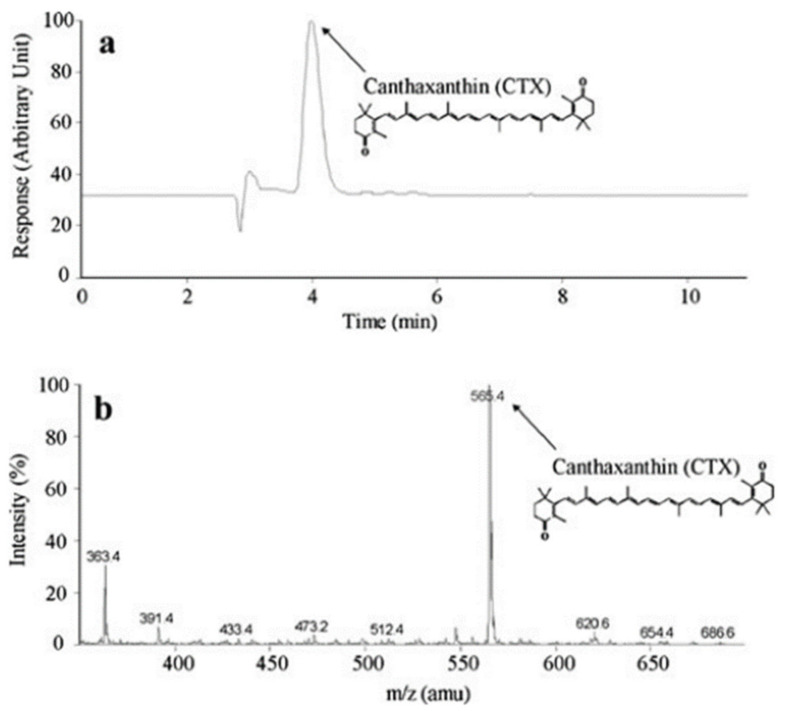
Typical HPLC chromatogram (**a**) and atmospheric pressure chemical ionisation mass spectra performed in positive ion condition (**b**) obtained analysing the biomass produced by *D. natronolimnaea* HS-1.

Canthaxanthin was obtained by biofermentation using D. natronolimnaea HS-1 and an enzymatic hydrolysed molasses as substrate. The concentration of the obtained biomass in the grown medium was ~1.81 mg/L and contained ~1.01 mg/L of canthaxanthin, which was statistical equal to the concentration of biomass, indicating that canthaxanthin was the main carotenoid produced. The identification and quantification of carotenoid pigments contained in the biomass were carried out using the HPLC and the atmospheric pressure chemical ionisation tandem mass spectrometric analysis. The peak reported in Figure 1a confirms the presence of canthaxanthin as the main component in the obtained biomass. The most abundant fragment ion in the positive ion tandem mass spectrum was Canthaxanthin (C_40_H_22_O_2_) as detected at *m*/z 565.4, Figure 1b.

As previously reported, the concentration of canthaxanthin produced depended on the used concentration and kind of substrate, and using molasses increased when its concentration increased from 5 g/L to 25 g/L [22,23]. On the contrary, its production decreased in response to a further increase in molasses content from 25 g/L to 50 g/L. This result confirmed the existence of anaerobic and viscous conditions and ethanol synthesis because of the Crabtree effect at high concentrations of molasses under a batch process [23,24]. Reynders et al. [24,25] reported similar results in astaxanthin bioproduction using *Phaffia rhodozyma*; indeed its production was lowered when the glucose content increased. This fact might be due to the long-time exposure of dissolved carotenoids to oxygen, light, and microorganisms in the environment leading to an increased chance of oxidation or degradation of the dissolved carotenoids.

### 3.2. Vesicle Characterisation

A preformulation study was carried out to select the most homogenous dispersions containing stable and small vesicles. Different kinds and numbers of phospholipids and various water co-solvents and/or biopolymers were used, and their ability to incorporate an increasing amount of canthaxanthin was evaluated as well. Among all, soy lecithin (120 mg/mL) was selected as the phospholipid due to its direct plant origin (without transformation process) and the suitable characteristics of formed vesicles. A total of 20 mg/mL of canthaxanthin was selected as the most suitable loading concentration. Glycerol (30%) was used to partially substitute the water, and hyaluronan (0.1%) was also added to improve the homogeneity and stability of the resulting vesicles. The average diameter, polydispersity index, and surface charge of the vesicles were measured and compared as a function of the used components, using empty vesicles as reference (Table 2).

Empty vesicles were the smallest irrespective of the used components (~63 nm, *p* > 0.05 among the size of samples), were monodispersed (polydispersity index ~0.24), and highly negatively charged (~55 mV). The incorporation of canthaxanthin into liposomes and glycerosomes caused a slight increase in the mean diameter of the vesicle, which was ~71 nm (*p* > 0.05 between the mean diameter of two samples and *p* < 0.05 versus the mean diameter of other empty and loaded vesicles). The incorporation of canthaxanthin into hyalurosomes addressed a further increase in mean diameter up to ~87 nm (*p* < 0.05 versus the mean diameter of other vesicles). The loading effect was negligible in glycerohyalurosomes with their size being similar to that of empty vesicles (~63 nm, *p* > 0.05 versus the mean diameter of empty vesicles). The vesicle enlargement may be related to the intercalation of canthaxanthin inside the bilayer, which allowed a slightly modification of phospholipid assembling. The simultaneous presence of glycerol and hyaluronan deleted this effect. The surface charge of all the canthaxanthin-loaded vesicles was ~−50 mV, similar to that of the corresponding empty vesicles, indicating that it was mostly intercalated inside the bilayer and not located in the vesicle surface. The amount of canthaxanthin effectively incorporated in glycerosomes, hyalurosomes, and glycerohyalurosomes was higher (~78%, *p* > 0.05 among the values of this group) than that in liposomes (~57%), confirming that glycerol and sodium hyaluronate favoured the loading of canthaxanthin.

**Figure 2:**
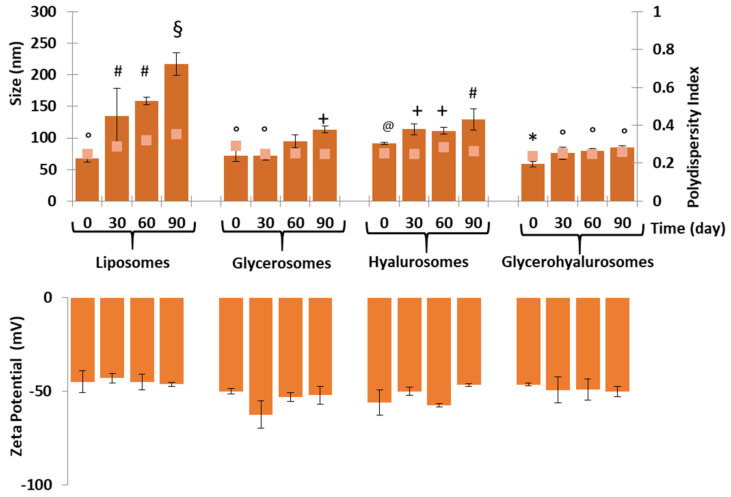
Mean diameter, polydispersity index, and zeta potential of liposomes, glycerosomes, hyalurosomes, and glycerohyalurosomes measured during 90 days of storage at 25 °C. The mean values ± standard deviations (error bars) are reported (*n* = 6). Each symbol (*, °, ^+^, ^§^, ^@^, ^#^) indicates the same value (*p* > 0.05 versus others).

The long-term stability of vesicles was evaluated by storing them at 25 °C in the dark for three months. Their size, polydispersity index, and zeta potential were measured at scheduled times (Figure 2). The mean diameter and polydisperity index of canthaxanthin-loaded liposomes increased progressively during the storage period. The mean diameter doubled after three months (~210 nm), while that of the other vesicles remained almost constant, and only a slight increase was observed for the size of glycerosomes and hyalusomes at 60 days and 90 days, respectively. After one month, the mean diameter of glycerohyalurosomes slightly increased up to 75 nm and then remined constant along with the polydispersity index and the surface charge, denoting a positive contribution of the combination of glycerol and sodium hyaluronate in the assembly and stability of the vesicle bilayer.

### 3.3. Evaluation of the Antioxidant Activity of Formulations

Canthaxanthin is a well-known antioxidant molecule. The free radical scavenging activity of the formulations was assessed by means of the DPPH colorimetric assay. The antioxidant activity in the ethanolic solution (20 mg/mL) was 51% ± 1 and slightly increased up to 58% ± 1 after its loading into the vesicles. The result underlined the suitability of prepared vesicles; indeed, they did not negatively affect the antioxidant activity of canthaxanthin, on the contrary, they provided a synergic effect due to the antioxidant molecules naturally occurring in lecithin [22,23].

### 3.4. Inhibition of Nitric Oxide Generation in Cells

**Figure 3:**
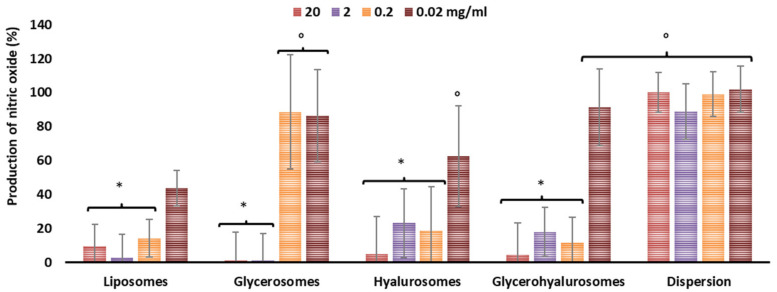
Expression of nitric oxide induced in cells with lipopolysaccharide and modulated by pre-treatment with canthaxanthin in dispersion or loaded in vesicles. Mean values ± standard deviations (error bars) are reported (*n* = 10). Each symbol (*, °) indicates the same value (*p* > 0.05 versus others).

Nitric oxide is expressed in large amounts in cells stimulated with lipopolysaccharide. This overexpression has been demonstrated to be associated in the pathogenesis of acute and chronic inflammatory conditions [26]. The capability of canthaxanthin formulations to reduce nitric oxide production in cells in vitro was measured (Figure 3). Lipopolysaccharide stimulated the production of nitric oxide in cells up to ~100%. Their pre-treatment with canthaxanthin in dispersion did not effectively counteract its release as the values were ~100%, irrespective of the used dilution (*p* > 0.05 among the values at different dilutions). The treatment of the inflamed cells with canthaxanthin loaded into vesicles inhibited the nitric oxide release in a dose- and composition-dependent manner. Indeed, any inhibition (~100% of nitric oxide, *p* > 0.05 versus the results obtained in untreated cells) was detected by using the higher dilution (concentration 0.02 μg/mL), except when liposomes were used, as the inhibition was ~40% (*p* < 0.05 versus others). When canthaxanthin was loaded in glycerosomes, hyalurosomes, and glycerohyalurosomes at lower dilutions (concentrations 20, 2, 0.2 μg/mL), the nitric oxide production was almost completely inhibited (nitric oxide produced <10%, *p* < 0.05 versus values of cells treated with vesicles at higher dilution), except using glycerosomes at an intermediate dilution (concentration 0.2 μg/mL), which was not effective.

### 3.5. Biocompatibility of Formulations

**Figure 4:**
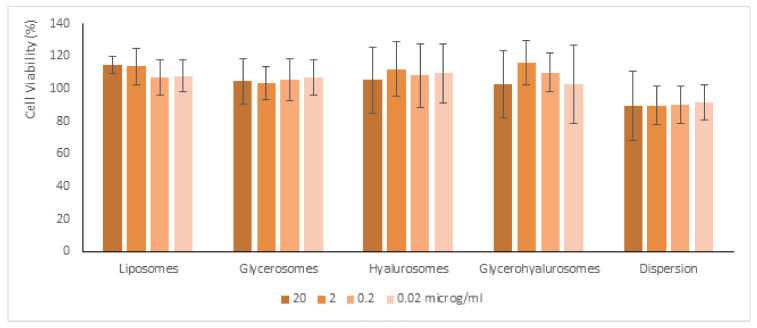
Cell viability of fibroblasts treated for 48 h with canthaxanthin in dispersion or loaded in vesicles diluted to reach 0.02 μg/mL, 0.2 μg/mL, 2 μg/mL, and 20 μg/mL of bioactive molecule. Data are reported as mean values (*n* = 9) ± standard deviations (error bars) of cell viability expressed as the percentage of untreated cells (100% of viability).

The most representative cells of the dermis are fibroblasts (3T3), which are mainly involved in skin repair. Given that, they were used to evaluate in vitro the potential cytotoxicity of samples (Figure 4). The cells were incubated for 48 h with formulations properly diluted with the medium to reach four different concentrations of canthaxanthin (0.02 μg/mL, 0.2 μg/mL, 2 μg/mL, and 20 μg/mL), and their viability was measured. The viability of 3T3 cells incubated with canthaxanthin in dispersion was ~89%, irrespective of the used dilution (*p* > 0.05 among the values obtained with different dilutions). The incubation with the canthaxanthin-loaded liposomes achieved similar values (~96%, *p* > 0.05 versus the values obtaining with dispersion). The incubation with canthaxanthin loaded in glycerosomes, hyalurosomes, and glycerohyalurosomes increased the cell viability up to ~110%, even if the values were not statistically different from that obtained using the dispersion (*p* > 0.05 among the viability values obtained using dispersion and vesicles). Thus, the results indicate that the loading of canthaxanthin in these vesicles can address a proliferative effect in fibroblasts [24,25,27].

### 3.6. Protective Effect of the Formulations against Damages Induced by Hydrogen Peroxide

**Figure 5:**
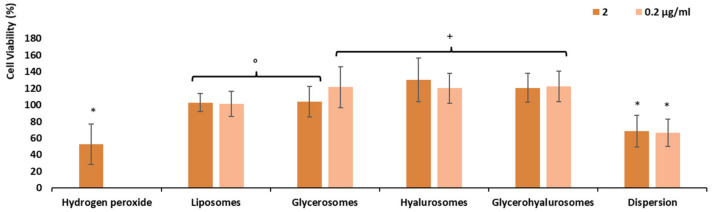
Viability of fibroblasts stressed with hydrogen peroxide and protected with canthaxanthin (2 μg/mL and 0.2 μg/mL) in dispersion or loaded in vesicles. Data are reported as mean values (*n* = 9) ± standard deviations (error bars) of cell viability expressed as the percentage of untreated cells (100% viability). Each symbol (*, °, ^+^) indicates the same value (*p* > 0.05 versus others).

Hydrogen peroxide can decrease the viability of human skin fibroblasts with a concurrent increase in generation of reactive oxygen species and cell apoptosis [28]. Many studies have indicated that high concentrations of hydrogen peroxide under pathological conditions may induce human skin degeneration and ageing [29]. Given that, the ability of canthaxanthin-loaded vesicles to counteract its toxic effect in fibroblasts was evaluated. Considering the biocompatibility of formulations at all the tested dilutions, only intermediated dilutions corresponding to 2 e 0.2 µg/mL of canthaxanthin were selected for these studies. As expected, the stress induced in cells with hydrogen peroxide led to a significant reduction of fibroblast viability (~53%, *p* < 0.05 versus values obtained using the vesicles) (Figure 5). The treatment of stressed cells with canthaxanthin in dispersion (2 e 0.2 µg/mL) did not induce an increased cell viability statistically different from that of cells stressed and untreated, irrespective of the used dilution (*p* > 0.05 versus values obtained with hydrogen peroxide). The treatment with canthaxanthin loaded in vesicles ensured a complete protection of fibroblasts, as the cell viability was ≥100%. The protection was formulation depending as the viability of cells treated with canthaxanthin (2 μg/mL) loaded in liposomes and glycerosomes was ~100% (*p* < 0.05 versus values obtained with cells protected with the dispersion), and that of cells treated with 0.2 μg/mL of canthaxanthin loaded in glycerosomes, hyalurosomes, and glycerohyalurosomes was ~120% (*p* < 0.05 versus other values). The loading improved the effectiveness of the payload probably favouring a better internalisation of it inside the cells and their membrane, which is widely damaged by reactive oxygen species [30].

### 3.7. In Vitro Scratch Assay

**Figure 6:**
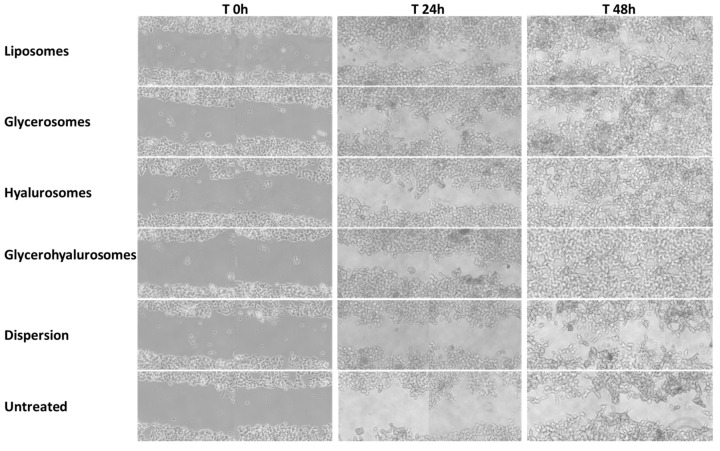
Representative images of wound closure in a monolayer of fibroblasts untreated or treated with canthaxanthin in dispersion or loaded in vesicles at 0 h, 24 h, and 48 h.

**Figure 7:**
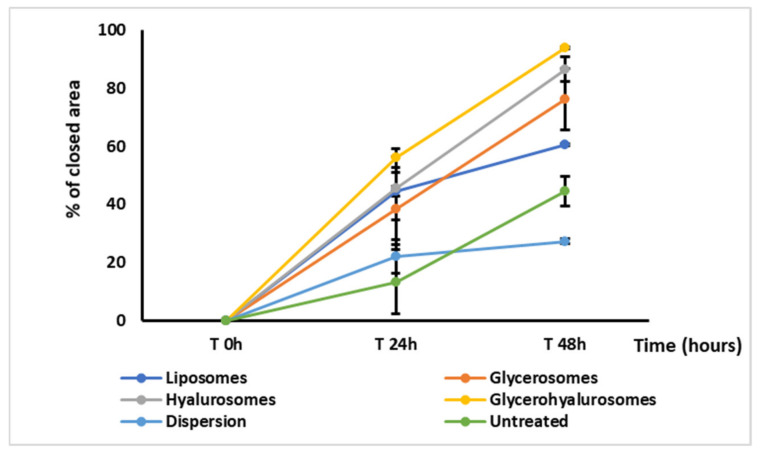
Percentage of wound closure in a monolayer of fibroblasts untreated or treated with canthaxanthin in dispersion or loaded in vesicles at 0 h, 24 h, and 48 h. Mean values ± standard deviations (error bars) are reported (*n* = 6).

An in vitro scratch assay was carried out on a cell monolayer of fibroblasts to evaluate the capability of canthaxanthin in dispersion or loaded in vesicles of favouring the wound closure promoting cell proliferation and migration (Figure 6 and Figure 7). The untreated cells, which predict the wound behaviour under physiological conditions, did not reach the complete closure of the wounded area at 48 h, as the percentage of closure was ~33%. A reduction in the scratched area was observed at 48 h (46% of closure) when cells were treated with canthaxanthin in dispersion, while an almost complete closure was obtained when the cells were treated with canthaxanthin loaded in vesicles, especially in hyalurosomes (87% of closure) and glycerohyalurosomes (94% of closure), revealing an optimal potential of these formulations in accelerating the healing of a wound performed in a cell monolayer.

## 4. Discussion

There is an increasing demand for carotenoids, such as canthaxanthin, due to their applications in the pharmaceutical, cosmeceutical, flavour food, and feed industries; however, the extraction and synthesis of these compounds can be expensive, technically challenging, and environmentally hazardous. The biotechnological production of canthaxanthin may provide an attractive alternative at the industrial level, which meets the growing demand to reduce costs and environmental pollution and avoid geographical and seasonal limitations [31,32]. An adequate formulation into appropriate nanocarriers can improve the other important limits to the actual use of canthaxanthin in commercial health-promoting products: instability and low availability in biological fluids [33,34]. In the present study, two strategies were combined to obtain green, scalable, and effective nanoformulations for the delivery of canthaxanthin in the skin: It was bioproduced using *D. natronolimnaea* and molasses as the inexpensive and renewable substrate in place of glucose, and it was loaded in natural-based vesicles prepared with soy lecithin, glycerol, hyaluronan, and water. The applied biotechnological methodologies addressed the microbial production of canthaxanthin in high yield by the valorisation of an agrifood byproduct and avoided the new glucose dissipation, as confirmed in previous studies [35]. According to the present results, Gharibzahedi et al. [19] previously used the molasses as a substrate to produce canthaxanthin and found that a low concentration of molasses (25 g L/L) caused an improvement of its synthesis, whereas high levels of this carbon substrate led to the inhibition of its production. The applied model was easy and permitted the scaling-up, control, and optimisation of the *D. natronolimnaea* culture process and can also be used as a guideline for similar microbial cultivation systems. The biotechnological production of canthaxanthin has been combined with the formulation of nanotechnological carriers, which previously revealed to be optimal carriers for skin delivery of natural molecules and carotenoids [34]. They are versatile and highly biocompatible systems capable of interacting with the cells thus effectively promoting the beneficial effects of the delivered molecule. To formulate green products, vesicles were obtained using soy lecithin, which is a phospholipid mixture from soy [36]. They were prepared applying an easy, repeatable, and low-dissipative method, which consists of the dispersion of all components in the water phase and direct sonication, thus avoiding the use of organic solvents [32,34]. Liposomes were enriched with glycerol and hyaluronan due to their natural origin, beneficial effect on the skin, and high biocompatibility [37]. The obtained vesicles confirmed to have promising characteristics for skin delivery as they were small and homogeneously dispersed and negatively charged, due to the negative group of phosphatidylcholines [38]. Canthaxanthin was incorporated inside the bilayer in a high amount as suggested by the unchanged zeta potential in empty vesicles. In vitro studies have been carried out by using fibroblasts, as the most representative cells of the skin. The results confirm the high biocompatibility of the formulations along with their in vitro beneficial effects. The incorporation of canthaxanthin into the vesicles improved its protective effect in counteracting the damages and death of cells injured by hydrogen peroxide, probably due to the vesicle’s ability to interact with fibroblasts and promote the internalisation of the bioactivity inside the cells. Similarly, vesicles, especially those enriched with hyaluronan, were capable of inhibiting to a better extent than the corresponding aqueous dispersion of canthaxanthin, the production of nitric oxide induced in vitro in macrophages using lipopolysaccharide [33,35,36,39]. The reduction of nitric oxide production promoted by the incorporation of canthaxanthin into the vesicles can be explained as the result of inhibition of iNOS protein expression. Indeed, the improved release of iNOS is generally responsible for the occurrence of many inflammatory disorders, in which inflammatory cytokines, such as IL-1β and TNF-α, are considered to be important. Even if the correct mechanism by which canthaxanthin is capable of counteracting the iNO production and preventing the inflammatory response is not clear yet, it can be assumed that thanks to its potent antioxidant activity, it may modify the redox state of the cell, which in turn may regulate directly or indirectly the expression of inflammatory cytokines [34,40]. However, more detailed and specific studies should be performed in vivo to confirm the anti-inflammatory effect of canthaxanthin.

The active interaction of phospholipid vesicles with the cells and their ability to favour payload internalisation, has been confirmed in previous studies. According to this, the treatment of a scratched area performed in a cell monolayer, with canthaxanthin-loaded vesicles, promoted the proliferation and migration of fibroblasts thus speeding up the closure of the wounded area in comparison with the aqueous dispersion of canthaxanthin. In particular, the combination of glycerol and hyaluronan seems to be the most promising as the complete closure during 48 h was detected only treating the cells with this formulation.

## 5. Conclusions

The demand of natural and safe products is increasing exponentially nowadays due to the awareness that a less polluted world ensures a better lifestyle. Even the synthesis of new molecules has been reduced in favour of the biotechnological production of both innovative and well-known bioactive molecules. In the present study, the effective and scalable bioproduction of canthaxanthin using *D. natronolimnaea* culture and molasses was confirmed as well as its efficacy in the skin when loaded into phospholipid vesicles, especially hyalurosomes and glycerohyalurosomes, which appeared as the most promising system for the treatment of skin damages.

## Figures and Tables

**Table 1:** Composition of vesicles.

	Canthaxanthin	Lecithin	Hyaluronan	Water	Glycerol
	mg/mL	mg/mL	mg/mL	mL	mL
Liposomes	20	120	-	1	-
Glycerosomes	20	120	-	0.7	0.3
Hyalurosomes	20	120	1	0.7	-
Glycerohyalurosomes	20	120	1	0.7	0.3

**Table 2:** Mean diameter (MD), polydispersity index (PI), zeta potential (ZP), and entrapment efficiency (EE) of empty and canthaxanthin-loaded vesicles. Mean values ± standard deviations are reported (*n* = 6). Each symbol (*, °, ^§^, ^#^) indicates the same value (*p* > 0.05 versus others).

	DM	(PI)	ZP (mV)	EE (%)
Empty liposomes	* 66 ± 2	0.25	−54 ± 3	−
Empty glycerosomes	* 65 ± 3	0.23	−49 ± 5	−
Empty hyalurosomes	* 64 ± 2	0.24	−57 ± 7	−
Empty glycerohyalurosomes	* 59 ± 3	0.23	−49 ± 3	
Canthaxanthin liposomes	° 72 ± 6	0.22	−48 ± 6	57 ± 5
Canthaxanthin glycerosomes	° 71 ± 5	0.26	−49 ± 2	^#^ 78 ± 3
Canthaxanthin hyalurosomes	^§^ 87 ± 11	0.25	−54 ± 9	^#^ 76 ± 6
Canthaxanthin glycerohyalurosomes	* 61 ± 4	0.22	−49 ± 5	^#^ 81 ± 4
